# Causal association between chloride intracellular channel protein 5 and Hashimoto thyroiditis: A Mendelian randomization study

**DOI:** 10.1097/MD.0000000000047347

**Published:** 2026-01-30

**Authors:** Juan Zhang, Yingnan Huang, Haibo Feng, Caixia Ding, Yang Yang, Zhigang Liu, Ruifang Sun

**Affiliations:** a Department of Pathology, Shaanxi Provincial Tumor Hospital, Xi’an Jiaotong University, Xi’an, Shaanxi, PR China; b Department of Head and Neck Surgery, Shaanxi Provincial Tumor Hospital, Xi’an Jiaotong University, Xi’an, Shaanxi, PR China; c School of Public Health, Shaanxi University of Chinese Medicine, Xianyang, Shaanxi, PR China; d Department of Thoracic Surgery, Shaanxi Provincial Tumor Hospital, Xi’an Jiaotong University, Xi’an, Shaanxi, PR China; e Department of Pathology, School of Basic Medical Sciences, Health Science Center, Xi’an Jiaotong University, Xi’an, Shaanxi, PR China.

**Keywords:** causal inference, chloride intracellular channel protein 5 (CLIC5), genome-wide association study (GWAS), Hashimoto thyroiditis, Mendelian randomization (MR)

## Abstract

This objective was to conduct Mendelian randomization (MR) analysis in order to investigate the potential causal relationship between chloride intracellular channel protein 5 (CLIC5) and Hashimoto thyroiditis (HT). Two separate MR analyses were performed, both using CLIC5 as the exposure variable and HT as the outcome. The first analysis utilized exposure data from genome-wide association study (GWAS) ID: prot-a-584 and outcome data from GWAS ID: ebi-a-GCST90018635. The second analysis used the same exposure data but sourced the outcome from ebi-a-GCST90018855. Four MR methods were applied in both analyses: MR Egger, weighted median, inverse variance weighted, and weighted mode. In both analyses, the inverse variance weighted method produced the most significant results, indicating a positive association between CLIC5 and HT. The first analysis estimated a causal effect of 0.4169 (*P* = .005344), while the second analysis estimated a slightly lower effect of 0.1196 (*P* = .0009933). The weighted median and weighted mode also suggested a positive association, although with lower statistical significance. The MR Egger method did not provide evidence for a causal effect in either analysis. Heterogeneity statistics did not indicate any significant evidence of heterogeneity, and the causal direction test strongly supported the hypothesis that CLIC5 caused HT. Horizontal pleiotropy was not detected. The consistency of the findings across different outcome databases enhances the credibility of the observed causal effect between CLIC5 and HT. Although the effect estimates varied slightly, these variations can be attributed to differences in the underlying genetic architectures captured by the respective GWAS studies.

## 1. Introduction

Autoimmune thyroid diseases, particularly Hashimoto thyroiditis (HT), represent a significant global health burden, affecting millions of individuals worldwide.^[1]^ These conditions are characterized by the immune system mistakenly attacking the thyroid gland, leading to inflammation, hormone imbalances, and potentially irreversible damage to the gland.^[2]^ Despite notable advances in our understanding of the genetic and environmental factors contributing to autoimmune thyroid diseases, the precise mechanisms underlying their pathogenesis remain poorly understood.

Intracellular chloride ion channel 5 (CLIC5) is a chloride ion channel expressed in multiple cell types and is involved in various physiological and pathological processes, including interactions with the cytoskeleton,^[3,4]^chloride ion transport,^[5]^ cell proliferation and differentiation,^[6]^ and maintenance of auditory and balance functions.^[4]^ As a member of the chloride channel family, CLIC5 has emerged as a candidate molecule that may play a crucial role in immune cell activation and regulation. For example, bioinformatics, qPCR, and IHC studies have found that the expression of CLIC5 in ovarian cancer is strongly positively correlated with M2 macrophage infiltration and negatively correlated with CD8 + T cell infiltration, suggesting its potential regulation of immune cells.^[7]^ Similarly, the analysis of 167 lung adenocarcinoma samples revealed that CLIC5 expression is closely related to the infiltration levels of many immune cells and immune marker sets in patients.^[8]^ Given that HT is an autoimmune disease and CLIC5 has the ability to regulate immune cells, it is plausible that CLIC5 could play a role in the regulation of HT. However, this has yet to be reported in the literature.

While no previous studies have suggested associations between genetic variants in CLIC5 and HT, establishing causality between CLIC5 and HT has been challenging due to the inherent limitations of observational studies, such as confounding and reverse causality. Mendelian randomization (MR), a statistical technique that leverages genetic variants as instrumental variables (IVs) to infer causality, offers a powerful approach to overcome these limitations and provide more robust evidence for causal relationships.^[9]^ MR analysis can improve the reliability of causal inference by using random allocation of genotypes to avoid the influence of confounding factors and reverse causal relationships. This method is based on genetic variation and uses random allocation of genotypes to study the causal relationship between exposure factors and diseases or other phenotypes, making it widely used in inferring the causal relationship between genes and disease states.

In this study, we aim to investigate the potential causal relationship between CLIC5 and HT using MR analysis. By leveraging summary statistics data from publicly available genome-wide association studies (GWAS), we will employ multiple MR methods to estimate the causal effect of CLIC5 on HT. Our findings have the potential to shed new light on the underlying mechanisms of autoimmune thyroid diseases and inform the development of novel therapeutic strategies targeting CLIC5.

## 2. Methods

To investigate the potential causal relationship between CLIC5 and HT, we conducted 2 separate MR analyses, using summary statistics data sourced from publicly available databases. MR is a statistical technique that leverages genetic variants as IVs to infer causality between an exposure and an outcome, thereby mitigating potential confounding factors commonly encountered in observational studies.

### 2.1. Data sources

For the exposure variable, CLIC5, we obtained summary statistics from a database identified by the unique identifier prot-a-584. These data encompassed effect estimates (beta coefficients) and standard errors (SEs) for genetic variants robustly associated with CLIC5 levels or expression. The outcome variable, HT, was sourced from 2 different GWAS databases: ebi-a-GCST90018635 for the first analysis and ebi-a-GCST90018855 for the second analysis (as detailed in Table 1). Both databases provided effect estimates and SEs for genetic variants associated with HT.

**Table 1:** Information from 3 datasets.

	Dataset	Population	Sex	Sample size	Number of SNP	Author
CLIC5	prot-a-584	European	M&F	3301	10,534,735	Sun BB
HT	ebi-a-GCST90018635	East Asian	NA	173,193	12,453,732	Sakaue S
	ebi-a-GCST90018855	European		395,640	24,146,037	Sakaue S

CLIC5 = chloride intracellular channel protein 5, HT = Hashimoto thyroiditis, SNP = single nucleotide polymorphisms.

### 2.2. Instrumental variable selection

Genetic variants were selected as IVs based on the following criteria: demonstrating a strong association with the exposure (CLIC5) at a genome-wide significance threshold (*P* < 5 × 10^−6^); ensuring independence from each other (linkage disequilibrium *R*^2^ < 0.001 within a 10,000-kb window to avoid collinearity); and having availability of effect estimates for both the exposure and the outcome. We performed clumping to guarantee that only a representative set of independent variants was included in the analysis. The exposure and outcome datasets were harmonized.

### 2.3. Mendelian randomization analysis

We employed 4 primary MR methods to estimate the causal effect of CLIC5 on HT.

#### 2.3.1. Inverse variance weighted method

This is the most commonly used MR method, which weights the effect estimates of individual IVs by the inverse of their variance. It operates under the assumption that all IVs are valid and that there is no pleiotropy (i.e., IVs affect the outcome only through the exposure).

#### 2.3.2. Weighted median method

A more robust method that estimates the causal effect using the weighted median of the effect estimates across IVs. It exhibits less sensitive to outliers and can tolerate up to 50% of the IVs being invalid due to pleiotropy.

#### 2.3.3. MR Egger regression

This method regresses the effect estimates of individual IVs on their associations with the exposure, enabling the detection of horizontal pleiotropy (i.e., IVs affecting the outcome through pathways other than the exposure).

#### 2.3.4. Weighted mode method

A method that identifies the most common effect estimate across IVs and assigns a weight based on the proportion of IVs supporting that estimate. It is particularly useful when the majority of IVs point in the same direction but there is some heterogeneity in the effect sizes.

### 2.4. Statistical analysis

For each MR method employed, we calculated the causal effect estimate (represented by the beta coefficient), the SE, and the corresponding *P*-value. Additionally, we conducted heterogeneity tests to evaluate the consistency of effect estimates across IVs. The Egger regression intercept was utilized to assess the presence of horizontal pleiotropy. Lastly, we performed a causal direction test to infer the direction of causality between the exposure and the outcome.

All 2-sample Mendelian randomization analyses were performed in September 2024 using summary statistics data from the IEU OpenGWAS project (formerly the MR Base platform). The analyses were conducted using the publicly available TwoSampleMR R package; during the study period, this was facilitated by the integrated online interface of the then-accessible MR Base platform. The complete data underlying this work are available from the IEU OpenGWAS repository (https://gwas.mrcieu.ac.uk/).

## 3. Results

The results of the 2 MR analyses, examining the potential causal relationship between CLIC5 and HT, are presented here. In both analyses, CLIC5 served as the exposure variable and HT as the outcome, with data sourced from different databases. Specifically, the first analysis used data for the exposure identified in a database with GWAS ID: prot-a-584 and the outcome from GWAS ID: ebi-a-GCST90018635 (East Asian population), while the second analysis utilized the same exposure but an outcome sourced from ebi-a-GCST90018855 (European population).

### 3.1. Causal effect of CLIC5 on HT

In the first analysis, using the outcome sourced from ebi-a-GCST90018635, 4 primary MR methods were applied: MR Egger, weighted median, inverse variance weighted (IVW), and weighted mode. The IVW method produced the most significant result, indicating a positive association between CLIC5 and HT with an estimated causal effect of 0.4169 (SE = 0.1497, *P* = .005344). The weighted median and weighted mode methods also suggested a positive association, albeit with lower statistical significance (*P* = .00931 and .03382, respectively). The MR Egger method did not provide evidence for a causal effect (*P* = .6986; Table 2).

**Table 2:** Mendelian randomization analysis of chloride intracellular channel protein 5 on hashimoto thyroiditis.

Method	ebi-a-GCST90018635				ebi-a-GCST90018855			
	nSNP	*b*	SE	*P* val	nSNP	*b*	SE	*P* val
MR Egger	8	0.2188	0.5385	.6986	15	0.1255	0.09629	.215
Weighted median	8	0.5236	0.2013	.00931	15	0.08047	0.05202	.1219
Inverse variance weighted	8	0.4169	0.1497	.005344	15	0.1196	0.03632	.0009933
Weighted mode	8	0.6386	0.2427	.03382	15	0.08463	0.08006	.3084

SNP = single nucleotide polymorphisms.

The second analysis, using the outcome sourced from ebi-a-GCST90018855, also demonstrated a positive association between CLIC5 and HT, albeit with slightly different effect estimates. The IVW method again yielded the most significant result, estimating a causal effect of 0.1196 (SE = 0.03632, *P* = .0009933). The weighted median and weighted mode methods provided less significant evidence of an association (*P* = .1219 and .3084, respectively), while the MR Egger method did not detect a causal effect (*P* = .215; Table 2).

### 3.2. Sensitivity analysis of MR

In the first analysis, the heterogeneity statistics derived from the IVW and MR Egger methods did not indicate any significant evidence of heterogeneity (with *Q*_*P*val = 0.6726 and 0.5762, respectively), suggesting consistency across the instruments used (Table 3). The causal direction test strongly supported the hypothesis that the exposure (CLIC5) caused the outcome (HT), with a directionality *P*-value of 1.71e^−45^ (Table 4). Furthermore, horizontal pleiotropy was not detected, as evidenced by the Egger regression intercept (0.054, *P* = .715; Table 5).

**Table 3:** Heterogeneity statistics.

Method	ebi-a-GCST90018635			ebi-a-GCST90018855		
	*Q*	*Q*_df	*Q*_*P*val	*Q*	*Q*_df	*Q*_*P*val
MR Egger	4.75	6	.5762	10.77	13	.6297
Inverse variance weighted	4.897	7	.6726	10.78	14	.7033

**Table 4:** Causal direction test.

	ebi-a-GCST90018635	ebi-a-GCST90018855
Variance explained in exposure	0.063	0.13
Variance explained in outcome	7.3e−05	5.5e−05
Inferred direction of causality	Exposure causes outcome	Exposure causes outcome
Directionality *P*-value	1.71e−45	1.2e−99

**Table 5:** Horizontal pleiotropy.

	ebi-a-GCST90018635	ebi-a-GCST90018855
Egger regression intercept	0.054	−0.0016
Standard error	0.14	0.024
Directionality *P*-value	.715	.948

In the second analysis, heterogeneity was not a concern in this analysis either, as indicated by the *Q*_*P*val from both the IVW and MR Egger methods (0.7033 and 0.6297, respectively; Table 3). The causal direction test similarly supported the exposure causing the outcome, with a directionality *P*-value of 1.2e^−99^ (Table 4). Horizontal pleiotropy was not detected, as evidenced by the Egger regression intercept (−0.0016, *P* = .948; Table 5).

**Figure 1.**
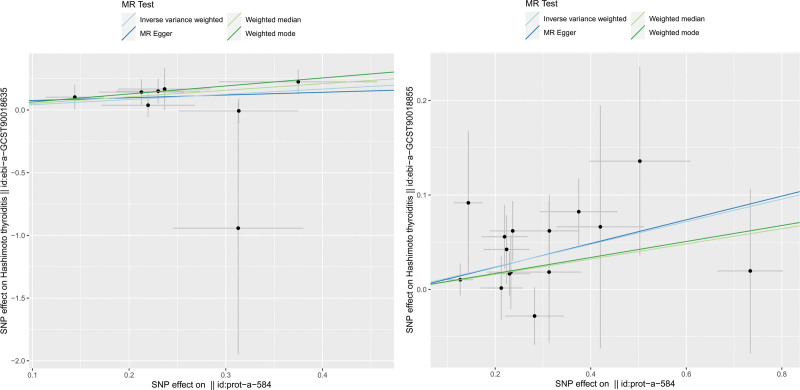
Scatter plot the genetic risks of chloride intracellular channel protein 5 on Hashimoto thyroiditis. MR = Mendelian randomization, SNP = single nucleotide polymorphisms.

**Figure 2.**
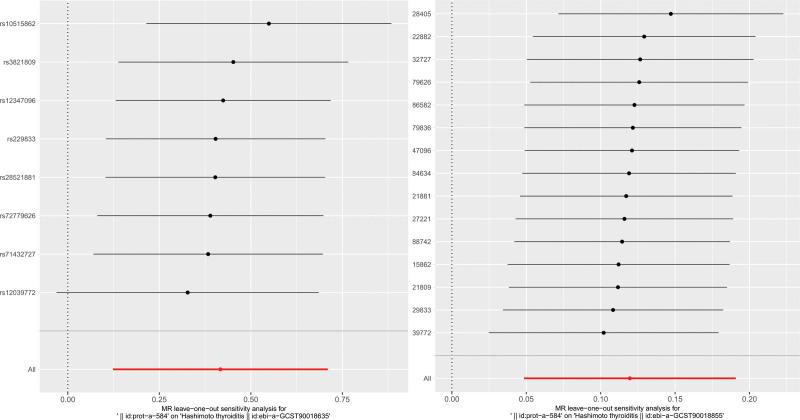
Leave-one-out tests of the genetic risks of the genetic risks of chloride intracellular channel protein 5 on Hashimoto thyroiditis. SNP = single nucleotide polymorphisms

The scatter plots and forest plots are presented in Figure 1 and Figure S1, Supplemental digital content, https://links.lww.com/MD/R281, respectively. The funnel plots were symmetrical (Fig. S2, Supplemental digital content, https://links.lww.com/MD/R281) and the leave-one-out method showed that no single SNP was substantially driving the association between CLIC5 and HT (Fig. 2).

Collectively, the consistency of the findings across different outcome databases strengthens the credibility of the observed causal effect. Although the effect estimates varied slightly, these can be attributed to differences in the underlying genetic architectures captured by the respective GWAS studies. Prospective studies with larger sample sizes and more comprehensive genetic data hold the promise of further refining these estimates.

## 4. Discussion

The results presented in this study provide compelling evidence for a potential causal relationship between CLIC5 and HT. Both analyses consistently suggest a causal relationship, with the IVW method offering the most robust evidence for this association in both instances. This discovery is novel and has not been previously reported. By employing MR analysis, we were able to mitigate some of the inherent limitations of observational studies, such as confounding and reverse causality, thereby enhancing the credibility of our conclusions.

Notably, the consistency of the results across 2 separate analyses using outcomes sourced from different GWAS databases (ebi-a-GCST90018635 and ebi-a-GCST90018855) reinforces the robustness of our findings. This consistency suggests that the observed causal effect is not driven by specific features of a single study or dataset. Furthermore, the IVW method consistently yielded the most significant results in both analyses, providing the strongest evidence for a positive association between CLIC5 and HT.

The lack of significant heterogeneity across the IVs used in our analyses is reassuring, as it indicates that the effect estimates are consistent and not influenced by outliers. This consistency is crucial for the validity of MR studies, as heterogeneity can suggest the presence of pleiotropy or other violations of the MR assumptions. The Egger regression intercepts did not provide evidence for horizontal pleiotropy, further supporting the validity of our findings. Additionally, the causal direction test strongly supports the hypothesis that CLIC5 causes HT, rather than the reverse. However, it is important to note that MR studies cannot definitively prove causality but rather provide evidence that is consistent with a causal interpretation.

Human genetic polymorphism plays a vital role in explaining human susceptibility and tolerance to diseases, as well as clinical phenotype diversity for HT.^[10]^ In a large-scale Swedish twin study, the concordance rates for HT in monozygotic twins and dizygotic twins were 0.29 and 0.1, respectively, with an estimated heritability of 0.64, highlighting the significance of both genetic and environmental factors in determining susceptibility.^[11]^ Furthermore, in a study from Croatia involving a confirmation cohort of 303 patients, 3 variants were identified as association with HT. Genetic risk score revealed that they accounted for 4.82% of the total genetic variance in HT.^[12]^ However, current research has not identified significant genetic polymorphisms that are specific and sensitive to HT. These all suggest that HT is influenced by gene polymorphism, which provides a clue for us to choose MR analysis.

HT is a prototypical organ-specific chronic inflammatory disease mediated by T cells, characterized by a significant infiltration of lymphocytes in the thyroid tissue. Cytokines derived from the lymphocyte infiltration play a crucial role in enhancing the thyroid cells’ capacity to release pro-inflammatory mediators, thereby amplifying and sustaining autoimmune responses.^[13]^ Therefore, correcting imbalanced immune function is the fundamental approach to treating HT. Chloride channels not only participate in the recruitment of immune cells but also play an important role in the activation of these cells,^[14]^ which may be implicated in immune cell function and autoimmune disease pathogenesis.

CLIC plays a pivotal role in the regulation of immune cells, influencing their function.^[7]^ Huang’s research found that the expression of CLICs is significantly positively correlated with 6 types of infiltrating immune cells (B cells, CD8 + T cells, CD4 + T cells, neutrophils, macrophages, and DCs). In the subgroup with higher levels of neutrophil infiltration, patients with low CLIC5 expression have a poorer prognosis; in the subgroup with highly infiltrated macrophages, patients with low CLIC5 expression had poorer overall survival.^[15]^ Although there are currently no reports on the role of CLIC in HT, given that CLIC influences immune function and HT is a chronic inflammatory disease, it can be inferred that alterations in CLIC directly impact the progression of HT. This study clearly provides solid theoretical support for revealing the relationship between CLIC and HT.

Several limitations of our study should be acknowledged. First, the sample sizes of the original GWAS studies may have limited our power to detect smaller causal effects. Future studies with larger sample sizes may help to refine our estimates and identify additional genetic variants that could be used as IVs. Second, while we attempted to mitigate the potential for pleiotropy through the use of multiple MR methods, we cannot completely rule out the possibility that some of the IVs may have influenced HT through pathways other than CLIC5. Third, our findings are based on genetic associations and may not fully capture the complex interplay between environmental factors and CLIC5 in the development of HT.

## 5. Conclusion

Our study provides evidence for a potential causal relationship between CLIC5 and HT. This finding has important implications for understanding the underlying mechanisms of autoimmune thyroid disease and may inform the development of novel therapeutic strategies targeting CLIC5. Future studies are needed to validate our findings in independent cohorts and to explore the biological pathways linking CLIC5 to HT.

## Acknowledgments

We gratefully acknowledge the contributions of the original GWAS studies and databases that made these data available for secondary analyses. We extend our thanks to Sakaue S for providing HT data (ebi-a-GCST90018855, ebi-a-GCST90018635), to Sun BB for providing the public data of Chloride intracellular channel protein 5 (proto-a-584), and also to the IEU OpenGWAS project team (formerly the MR Base project). The MR Base online platform, accessible at the time of this study, provided essential tools for the Mendelian randomization analyses.

## Author contributions

**Conceptualization:** Ruifang Sun, Zhigang Liu.

**Data curation:** Yingnan Huang, Yang Yang.

**Formal analysis:** Juan Zhang.

**Investigation:** Yang Yang.

**Methodology:** Yingnan Huang, Haibo Feng.

**Project administration:** Caixia Ding.

**Resources:** Haibo Feng.

**Visualization:** Caixia Ding.

**Writing – original draft:** Juan Zhang, Zhigang Liu.

**Writing – review & editing:** Ruifang Sun, Juan Zhang.

## Supplementary Material


