# Supplementary figures and images for: Causal association between chloride intracellular channel protein 5 and Hashimoto thyroiditis: A Mendelian randomization study
Source: Medicine (Baltimore). 2026 Jan 30;105(5):e47347. doi: 10.1097/MD.0000000000047347 (PMC12863814; doi:10.1097/MD.0000000000047347)

Fig. S1 Forest plot

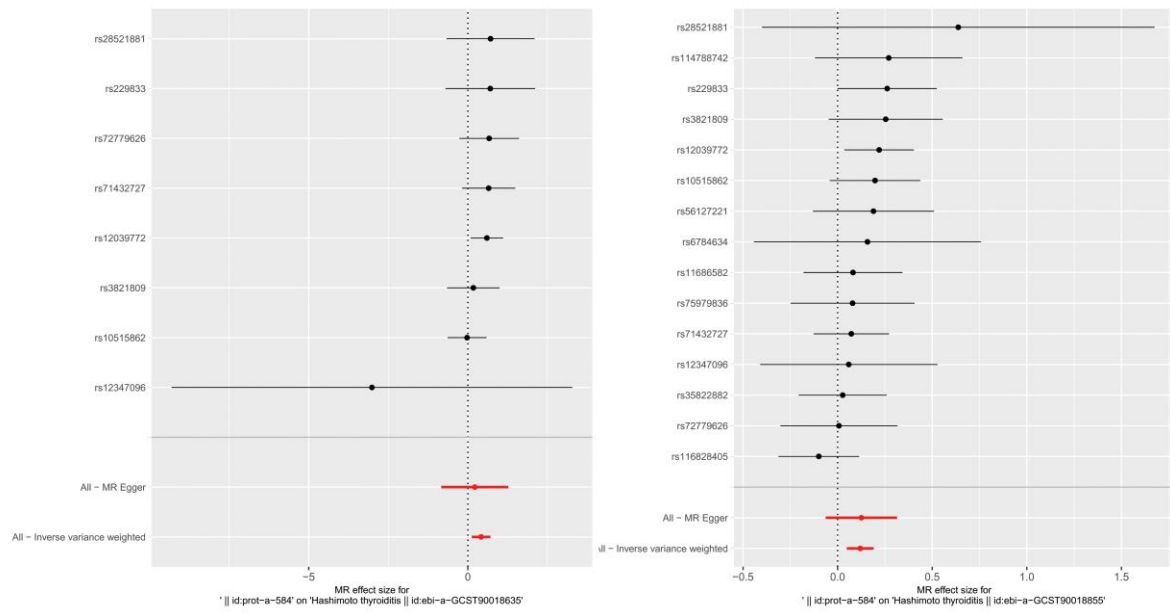

Fig. S2 Funnel plot

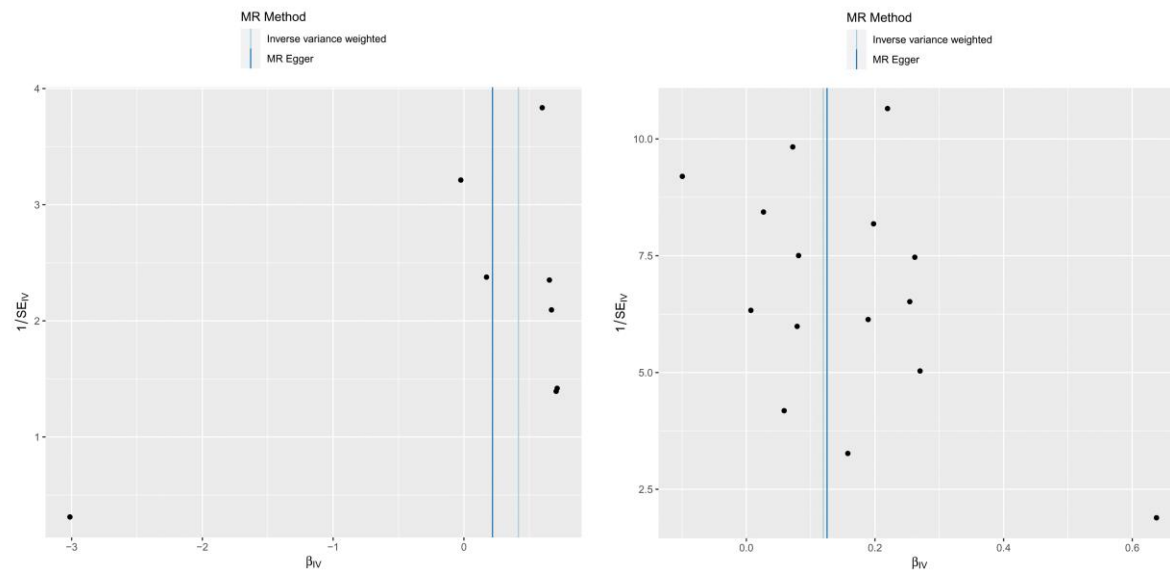

Supplement: Supplementary file 1 [file medi-105-e47347-s001.pdf]
